# OPEN SCIENCE AND TRANSPARENT RESEARCH PRACTICES: IMPLICATIONS FOR GERONTOLOGY

**DOI:** 10.1093/geroni/igz038.1479

**Published:** 2019-11-08

**Authors:** Derek M Isaacowitz, Jonathan W King

**Affiliations:** 1 Northeastern University, Boston, Massachusetts, United States; 2 National Institute on Aging, Bethesda, Maryland, United States

## Abstract

Scientists from many disciplines have recently suggested changes in research practices, with the goal of ensuring greater scientific integrity. Some suggestions have focused on reducing researcher degrees of freedom to extract significant findings from exploratory analyses, whereas others concern how best to power studies and analyze results. Yet others involve ensuring that other interested researchers can easily access study materials, code, and data, to help with re-analysis and/or replication. These changes are moving targets, with discussions and suggested practices ongoing. However, aging researchers have not yet been major participants in these discussions, and aging journals are just starting to consider open science policies. This symposium, sponsored by the GSA Publications Committee, will highlight transparency and open science practices that seem most relevant to aging researchers, discuss potential challenges to implementing them as well as reasons for doing so, and will consider how aging journals may implement these practices. Open science practices to be considered include: preregistration, open data, open materials and code, sample size justification and analytic tools for considering null effects. Presenters from a range of areas of aging research (lab, secondary data, qualitative) will show examples of open science practices in their work and will discuss concerns about, and challenges of, implementing them. Then, editorial team members will discuss the implications of these changes for aging journals. Finally, discussant Jon King will give NIA’s perspective on the importance of encouraging open science practices in the aging field.

